# Thermal enhancement and numerical solution of blood nanofluid flow through stenotic artery

**DOI:** 10.1038/s41598-022-20267-8

**Published:** 2022-10-19

**Authors:** Lubna Sarwar, Azad Hussain, Unai Fernandez-Gamiz, Sobia Akbar, Aysha Rehman, El-Sayed M. Sherif

**Affiliations:** 1 Department of Mathematics, University of Gujrat, Gujrat, 50700 Pakistan; 2 Nuclear Engineering and Fluid Mechanics Department, University of the Basque Country UPV/EHU, Nieves Cano 12, 01006 Vitoria-Gasteiz, Spain; 3 Department of Mechanical Engineering, College of Engineering, King Saud University, P.O. Box 800, Al-Riyadh, 11421 Saudi Arabia

**Keywords:** Biophysics, Applied mathematics

## Abstract

The blood flow through stenotic artery is one of the important research area in computational fluid mechanics due to its application in biomedicine. Aim of this research work is to investigate the impact of nanoparticles on the characteristics of human blood flow in a stenosed blood artery. In under consideration problem Newtonian fluid is assumed as human blood. Newtonian fluid flows through large blood vessels (more than 300 μm). The constitutive equations together with the boundary conditions are diminished to non-dimensional form by using boundary layer approximation and similarity transfiguration to attain the solution of velocity and temperature distribution of blood flow through arterial stenosis numerically with the help of Matlab bvp4c. The results for physical quantities at cylindrical surface are calculated and their effects are also presented through tables. The heat transfer rate increases throughout the stenosed artery with the concentration of copper nanoparticle. Velocity curve decreases by increasing the values of flow parameter and nanoparticle volume fraction. Temperature curve increases due to increase in the values of nanoparticle volume fraction and decrease in Prandtl number.

## Introduction

Today, due to modern lifestyle, smoking, high level of blood cholesterol and possibly a genetic problem we can see that arteries are obstructed. In arterial system the most common disease is hardening and contraction of the walls of blood vessels. In medical sciences it is known as arterial stenosis. Stenosis arteries are a constriction or narrowing of inner surface of arteries which consequently reduced the fluid flow passing to the other organs and tissues. The flow of blood in arterial stenosis is very significant discussion for the understanding of circulatory disorders due to the fact that main cause of many cardiovascular diseases are associated to the mechanical behavior of the blood vessel walls and nature of blood movement.

Biological fluids demonstrate all important information for the monitoring and diagnosis of numerous diseases, as well as for the foundation of the correct treatment. Examples of biological fluids involve blood; lymph, which is produced by the filtration of blood plasma through tissues; synovial fluid in joints; and the vitreous fluid of the eye. One of the important biofluid is blood, just like other bio-fluids. Experientially, it has been demonstrated that blood is the mixture of white blood cells (WBCs), red blood cells (RBCs) and platelets. Blood follows Newtonian nature when flowing through larger area arteries i.e., when the flow shear rate is high and is treated as non-Newtonian when flowing through area of smaller arteries, veins and in the downstream of the stenosis i.e., when the shear rate is below. The most earliest and basic paper about blood flow is of Ref.^1^ in which he proposed that the boundary asymmetry can be a major element in the progression and development of arterial disease. Many other researcher^2–6^ also analyzed the mathematical models of blood flow under different effects.

The latest and expanded field in the evolution of diagnostics and therapeutics is blood-mediated nanoparticle delivery. To increase function of nanoparticles in biological systems, their surface chemistry, size and shape can be managed. This enables blood clearance profile, transition of immune system interactions and reciprocity with target cells thereby supporting efficacious delivery of contents inside tissues or cells. Heat conduction ability of fluids like blood, water, ethylene glycol, engine oil and polymer solutions is low in the comparison of solids. Thermal conductivity of these fluids enhances by mixing the solid particles of higher thermal conductivity to these fluids. Sheikholeslami and Ebrahimpour^7^ studied the addition of nanoparticles to enhance the thermal ability of linear Fresnel solar system. Sheikholeslami et al.^8^ analyzed thermal behavior for parabolic solar system with addition of nanofluid. Awais et al.^9^ analyzed the addition of gyrotactic effects in bioconvection to stabilize the nanoparticles.

For different biomedical uses a large number of nanoparticles have been increased and some of them have shown great potential in imaging and treatment of diseases^10–18^. These are water, oils, lubricants, organic liquids (e.g., refrigerants, ethylene, triethylene glycols, etc.), biofluids, and polymeric mixture. The nanoparticles used in nanofluids are typically formed of metal oxides (silica, titania, zirconia and alumina), metal nitrides (SiN, AIN), metal carbides (SiC), chemically stable metals (copper, gold, aluminum), different types of carbon (graphite, fullerene, carbon nanotubes, diamond), and functionalization of nanoparticles. It is not a simple liquid–solid mixture; the collection of freely steady suspension for long duration without resulting in any chemical changings in the base fluid is the most important criterion of nanofluid.

Awan et al.^19^ presented the non-Newtonian fluid flow study through parallel plates by adding nanofluid and radiation effects. Qureshi et al.^20^ described the magnetic field effects during the peristaltic nanofluid flow through flexible tube. Awan et al.^21^ analyzed nanofluid flow with heat transfer, radiation and magnetohydrodynamic effects and obtained solution numerically by using backward difference solver. Awan et al.^22^ described heat transfer rate and entropy generation peristaltic fluid flow by adding nanoparticles and magnetic effects. Different researchers^9,23–33^ paid attention to mathematically analyze the fluid flow problems under addition of nanoparticles and different effects to enhance the heat transfer rate. Awais et al.^34^ investigated the impact of heat transportation over porous disk and calculated their solution numerically. Hussain et al.^35^ discussed the applications of nanoparticles through diseased artery. Samad Khan et al.^36^ studied non-Newtonian nanofluid flow through stretching surface and computed the heat dissipation by the phenomenon of joule heating and viscous dissipation. Awais et al.^37^ presented solution methodology based on artificial neural network for MHD non-Newtonian fluid flow problem through stretching sheet. Awais et al.^38^ discussed peristaltic motion with magnetic field and entropy generation with addition of copper as nanoparticle and water as base fluid.

With the above motivation, an attempt to develop a mathematical model to investigate the properties of the blood flow in the presence of arterial stenosis with the addition of nanoparticles is made. The nanofluid is considered as a mixture of copper and base fluid blood. Equations that govern the problem are solved and solution is attained numerically. Results for velocity and temperature profile are obtained by using MATLAB software. The physical features of distinct emerging parameters have been explained by plotting the graphs.

## Physical modeling

In under discussion problem we assumed that incompressible two dimensional flow of blood behaves as Newtonian fluid through stenosed artery of $\frac{{L}_{0}}{2}$ length and the flow field is steady. The coordinates are chosen in this manner that fluid flow towards $x-axis$ and $r-axis$ is considered normal to the fluid flow.

Consider two dimensional flow of blood in the presence of stenosed region of cosine shape constraint having unblocked area width ${2R}_{0}$, radius of the artery is $R\left(x\right)$ and the maximum stenosed region height is λ. Profile for stenosed region is chosen as

1 $$ \begin{array}{*{20}l} {R\left( x \right) = R_{0} - \frac{{\uplambda }}{2}\left( {1 + \cos \left( {\frac{4\pi x}{{L_{0} }}} \right)} \right),} \hfill & { - \frac{{L_{0} }}{4} < x < { }\frac{{L_{0} }}{4}} \hfill \\ {R\left( x \right) = R_{0} } \hfill & {Otherwise.} \hfill \\ \end{array} $$

With all above supposition, the steady boundary layer equations governing the flow and transfer of heat for Newtonian nanofluid are described as^39,40^:

2 $$\frac{\partial (ru)}{\partial x}+\frac{\partial (rv)}{\partial r}=0,$$

3 $$\left(u\frac{\partial }{\partial x}+v\frac{\partial }{\partial r}\right)u=\frac{{\upmu }_{nf}}{{\uprho }_{nf}}\frac{\partial }{r\partial r}\left(r\frac{\partial u}{\partial r}\right),$$

4 $$\left(u\frac{\partial }{\partial x}+v\frac{\partial }{\partial r}\right)T=\frac{{\mathrm{k}}_{nf}}{{(\uprho {C}_{p})}_{nf}}\frac{\partial }{r\partial r}\left(r\frac{\partial T}{\partial r}\right),$$

together with boundary conditions

5 $$\left.\begin{array}{l}u=0, v=0\, \text{and}\, T={T}_{1}\, \text{at}\, r=R,\\ \frac{\partial u}{\partial r}=0, \frac{\partial T}{\partial r}=0 \, \text{at}\,r=0.\end{array}\right\},$$

where viscosity is ${\upmu }_{nf}$ and ${(\uprho {C}_{p})}_{nf}$ represents the heat capacity of fluid and density is ${\uprho }_{nf}$ which are described in Tables 1 and 2.

**Table 1:** Thermophysical properties of nanofluid^41^.

Properties	Nanofluid
Density	${\rho }_{nf}={\rho }_{f}\left(\left(1-\phi \right)+\phi \frac{{\rho }_{s}}{{\rho }_{f}}\right)$
Viscosity	${\mu }_{nf}=\frac{{\mu }_{f}}{{\left(1-\phi \right)}^{2.5}}$
Heat capacity	${(\rho {C}_{p})}_{nf}={(\rho {C}_{p})}_{f}\left(\left(1-\phi \right)+\phi \frac{{(\rho {C}_{p})}_{s}}{{(\rho {C}_{p})}_{f}}\right)$
Thermal conductivity	$\frac{{k}_{nf}}{{k}_{f}}=\frac{{k}_{s}+2{k}_{bf}-2\phi \left({k}_{bf}-{k}_{s}\right)}{{k}_{s}+2{k}_{bf}+\phi \left({k}_{bf}-{k}_{s}\right)}$

**Table 2:** Base fluid (blood) and nanoparticles experimental values^42^.

Properties	Blood	Cu
$\rho (\mathrm{kg}/{\mathrm{m}}^{3})$	1063	8933
${C}_{p}({\mathrm{Jkg}}^{-1}{\mathrm{K}}^{-1})$	3594	385
$k$(${{\mathrm{Wm}}^{-1}\mathrm{K}}^{-1}$)	0.492	400

The continuity Eq. (1) can be satisfied by introducing stream function $\psi$ for $u$ and $v$ such that

6 $$u={r}^{-1}\partial \psi /\partial r, v={-r}^{-1}\partial \psi /\partial x .$$

Then Eqs. (3) and (4) becomes

7 $$\frac{1}{r}\frac{\partial \psi }{\partial r}\frac{\partial }{\partial x}\left(\frac{1}{r}\frac{\partial \psi }{\partial r}\right)-\frac{1}{r}\frac{\partial \psi }{\partial x}\frac{\partial }{\partial r}\left(\frac{1}{r}\frac{\partial \psi }{\partial x}\right)=\frac{{\upmu }_{nf}}{{\uprho }_{nf}}\frac{\partial }{r\partial r}\left(\frac{{\partial }^{2}\psi }{\partial {r}^{2}}-\frac{1}{r}\frac{\partial \psi }{\partial r}\right),$$

8 $$\left( \frac{1}{r}\frac{\partial \psi }{\partial r}\right)\frac{\partial T}{\partial x}-\left(\frac{1}{r}\frac{\partial \psi }{\partial x}\right)\frac{\partial T}{\partial r}=\frac{{\mathrm{k}}_{nf}}{{\left(\uprho {C}_{p}\right)}_{nf}}\frac{\partial }{r\partial r}\left(r\frac{\partial T}{\partial r}\right).$$

To transform the Eqs. (7) and (8) into ordinary differential equations, we have assumed the following transfiguration

9 $$u=\frac{{u}_{0}x}{{L}_{0}}{F}^{^{\prime}}\left(\eta \right), v=-\frac{R}{r}\sqrt{\frac{{u}_{0}{\nu }_{f}}{{L}_{0}}}F\left(\eta \right), \eta =\frac{{r}^{2}-{R}^{2}}{2R}\sqrt{\frac{{u}_{0}}{{{\nu }_{f}L}_{0}}} , \theta \left(\eta \right)=\frac{T-{T}_{0}}{{T}_{1}-{T}_{0}},$$

$$\psi =\sqrt{\frac{{u}_{0}{x}^{2}{\nu }_{f}}{{L}_{0}}}RF\left(\eta \right),$$

where $x=\frac{\widetilde{x}}{{L}_{0}}$ and after applying similarity transformation the Eqs. (7) and (8) finally becomes:

10 $$\frac{1}{{\left(1-\phi \right)}^{2.5}\left(\left(1-\phi \right)+\phi \frac{{\rho }_{s}}{{\rho }_{f}}\right)}\left[\left(1+2\gamma \eta \right){F}^{{^{\prime\prime\prime}}}+2\gamma F{^{\prime\prime}}\right]+F{F}^{{^{\prime\prime}}}-{{F}^{{\prime}}}^{2}=0,$$

11 $$\frac{{k}_{s}+2{k}_{bf}-2\phi \left({k}_{bf}-{k}_{s}\right)}{Pr\left({k}_{s}+2{k}_{bf}+\phi \left({k}_{bf}-{k}_{s}\right)\right)\left(\left(1-\phi \right)+\phi \frac{{\left(\rho {C}_{p}\right)}_{s}}{{\left(\rho {C}_{p}\right)}_{f}}\right)}\left[\left(1+2\gamma \eta \right){\theta }^{{{\prime\prime}}}+2\gamma {\theta }^{{\prime}}\right] +F{\theta }^{{\prime}}-{F}^{^{\prime}}\theta =0.$$

The non-dimensional form of Eq. (1) is

12 $$  \begin{array}{*{20}l} {f = 1 - \frac{\epsilon}{2}\left( {1 + \cos \left( {4\pi \tilde{x}} \right)} \right),} \hfill & { - \frac{1}{4} < \tilde{x} < { }\frac{1}{4}} \hfill \\ {f = 1} \hfill & {Otherwise,} \hfill \\ \end{array} $$

where $f=\frac{R(x)}{{R}_{0}}$ and $\epsilon =\frac{\uplambda }{{R}_{0}}$ is the non-dimensional measure of stenosis in reference artery.

The non-dimensional form of boundary conditions on stenosed artery are

13 $$ \begin{gathered} F\left( 0 \right) = 0, F^{^{\prime}} \left( 0 \right) = 0, \theta \left( 0 \right) = 1 \;{\text{at}} \;\eta = 0, \hfill \\ F^{\prime\prime}\left( \eta \right) = 0, \theta ^{\prime}\left( \eta \right) = 0 \; {\text{at}}\; \eta = f. \hfill \\ \end{gathered} $$

In the above equation non dimensional parameters are Prandtl number $Pr={k}_{f}/{(\mu {C}_{p})}_{f},$ flow parameter $\gamma =\sqrt{{\nu }_{f}{L}_{0}/{u}_{0}{R}^{2}}$ and $\phi$ represented solid nanoparticle volume fraction.

The significant quantities i.e., heat transfer coefficient $N{u}_{x}$ and skin friction coefficient ${C}_{f}$ of flow field are described:

14 $${C}_{f}=\frac{{\tau }_{w}}{\frac{1}{2}{\rho }_{f}{U}_{w}^{2}},$$

15 $$Nu=\frac{x{q}_{w}}{{k}_{f}({T}_{w}-{T}_{\infty })},$$

where shear stress ${\tau }_{w}$ is

16 $${\tau }_{w}={\mu }_{nf}{\left.\frac{\partial u}{\partial r}\right|}_{r=R},$$

and heat flux is

17 $${q}_{w}={-k}_{nf}{\left.\frac{\partial T}{\partial r}\right|}_{r=R},$$

Non-dimensional form of Eqs. (14) and (15) takes the form

18 $$R{e}_{x}^{1/2}{C}_{f}=\frac{1}{{\left(1-\phi \right)}^{2.5}}{F}^{{^{\prime}}{^{\prime}}}\left(0\right),$$

19 $$R{e}_{x}^{-1/2}N{u}_{x}=-\frac{{k}_{nf}}{{k}_{f}}{\theta }^{^{\prime}}\left(0\right),$$

where local Reynolds number represented by $R{e}_{x}^{-1/2}$.

## Numerical solution

The Eqs. (7) and (8) having nonlinearity are changed into ODE’s with the association of appropriate conversions. Their solution is obtained numerically by using bvp4c in MATLAB. Bvp4c is a finite difference code that uses the three stage Lobatto IIIa formula. Result for temperature and velocity curves are plotted to show the impact of distinct values of non-dimensional quantities.

## Graphical results and discussion

**Figure 1:**
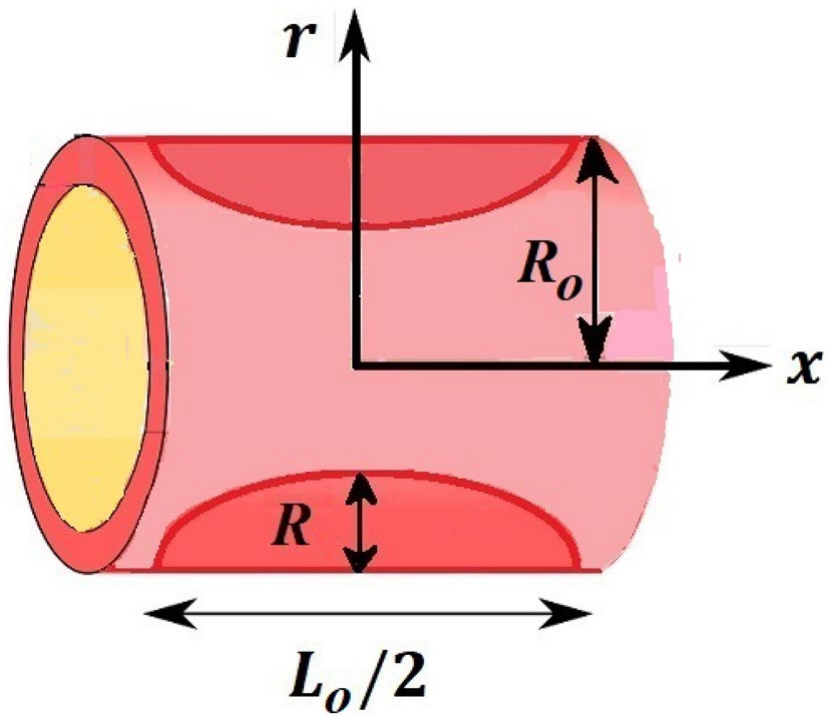
Geometrical structure of artery with stenosis.

**Figure 2:**
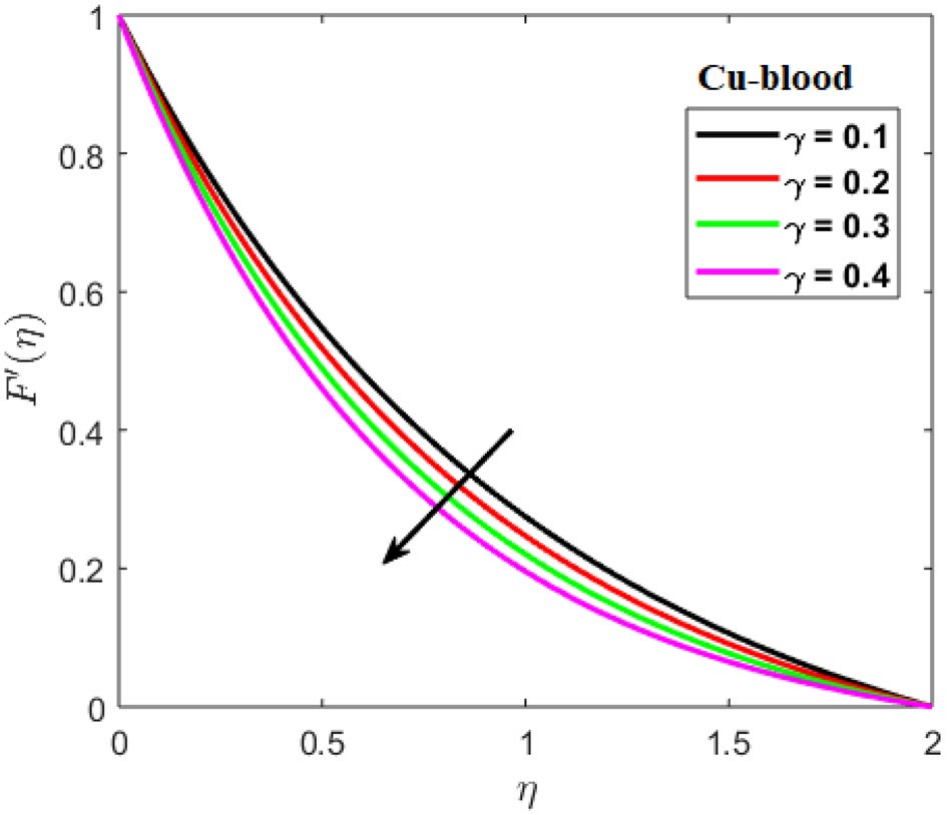
Results of $\gamma$ on velocity.

**Figure 3:**
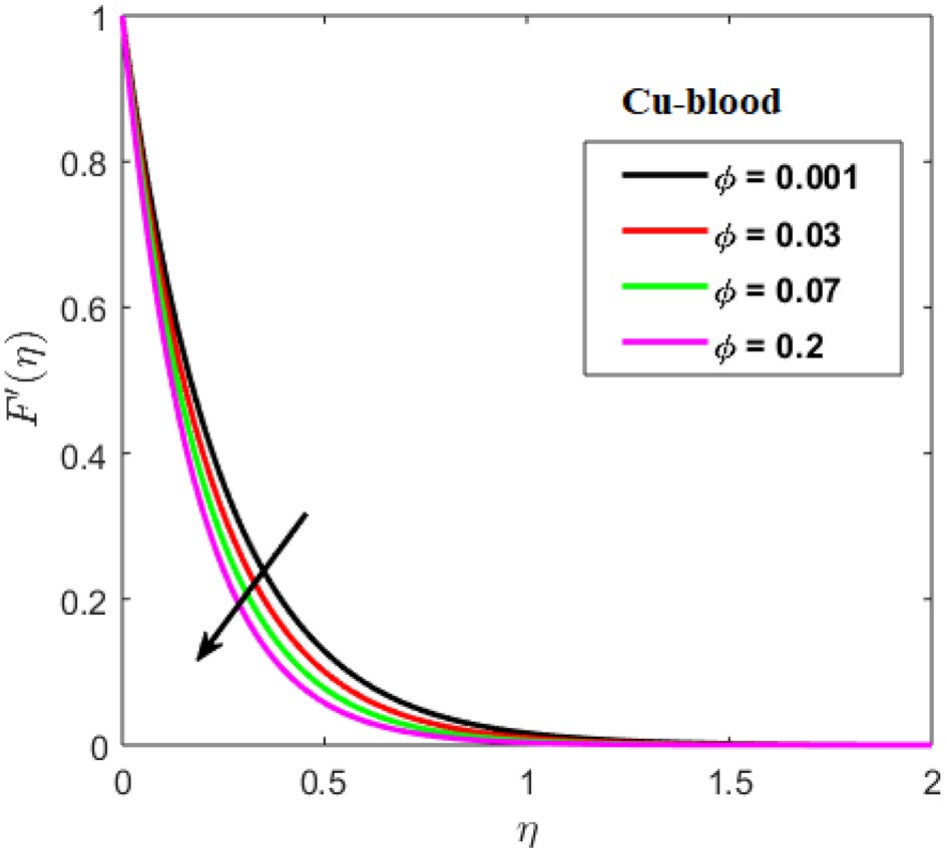
Results of $\phi$ on velocity.

**Figure 4:**
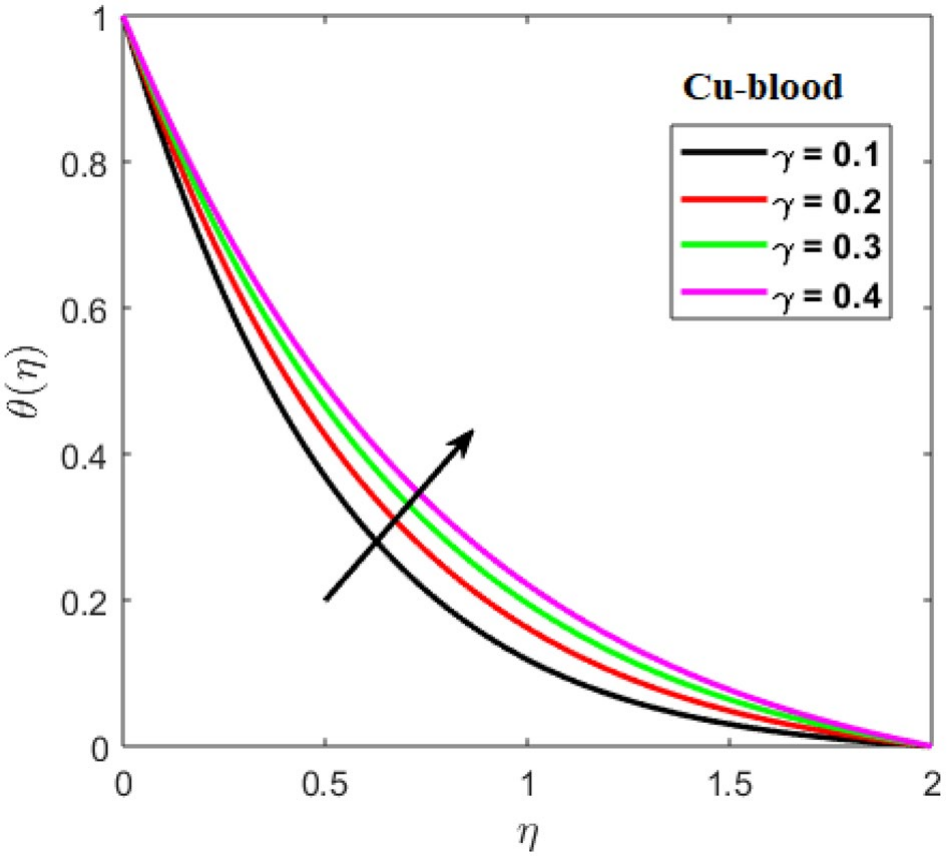
Impact of $\gamma$ on temperature.

**Figure 5:**
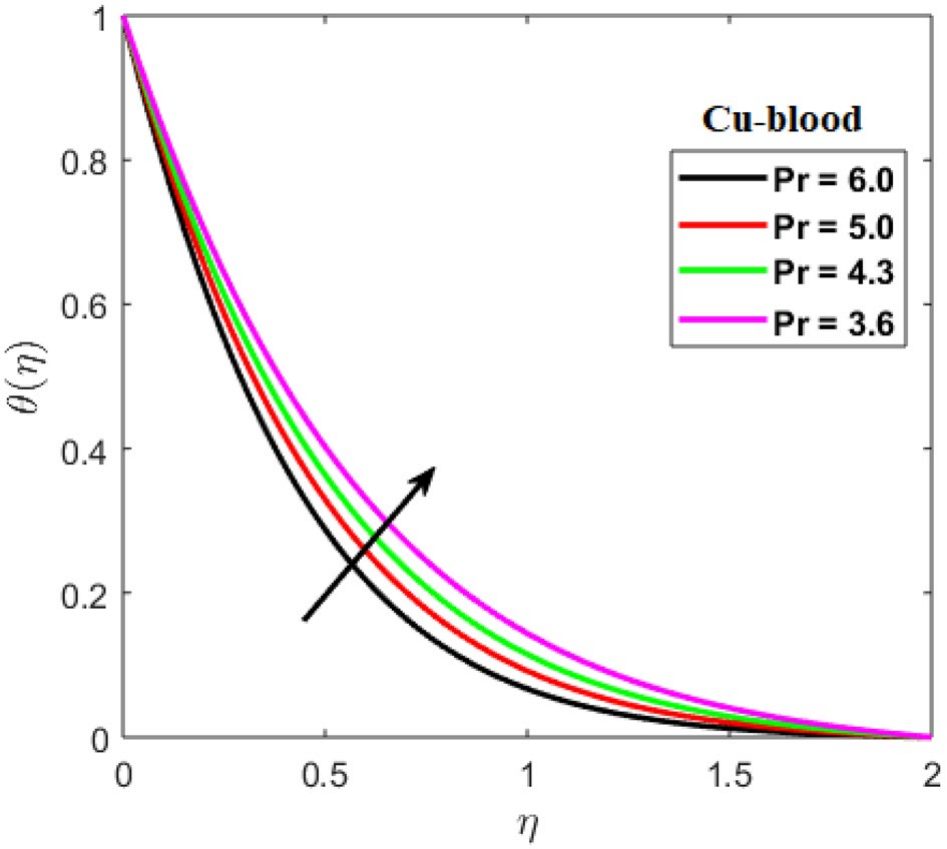
Profile of $Pr$ for temperature.

**Figure 6:**
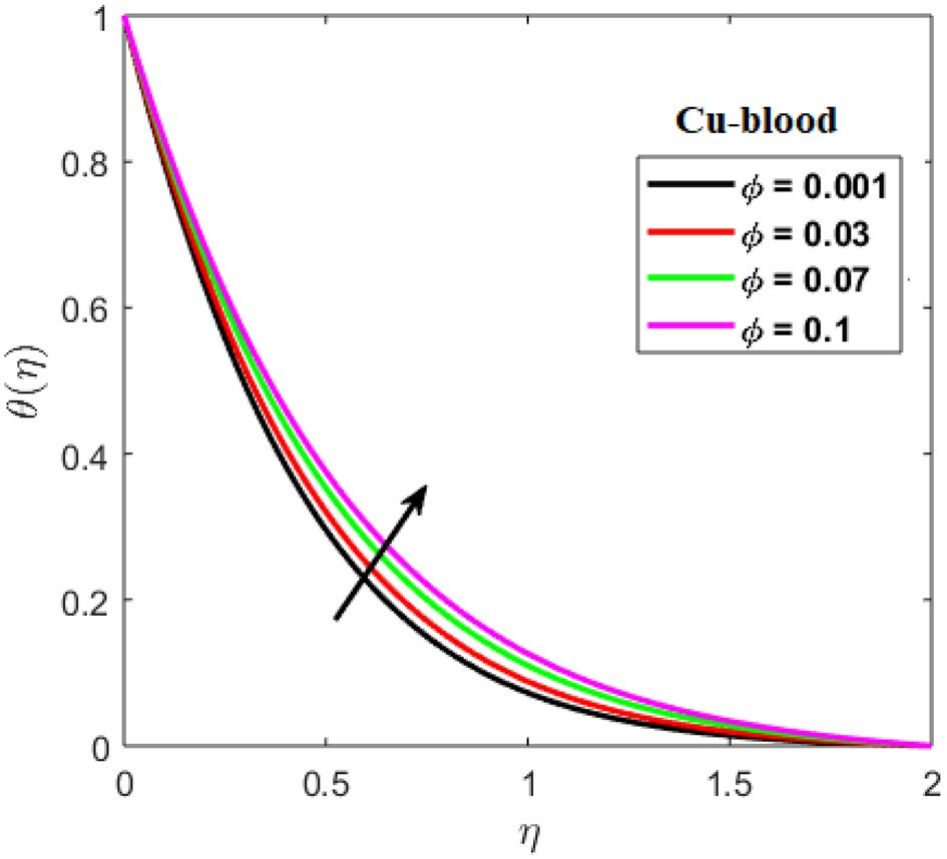
Results of $\phi$ on temperature.

**Figure 7:**
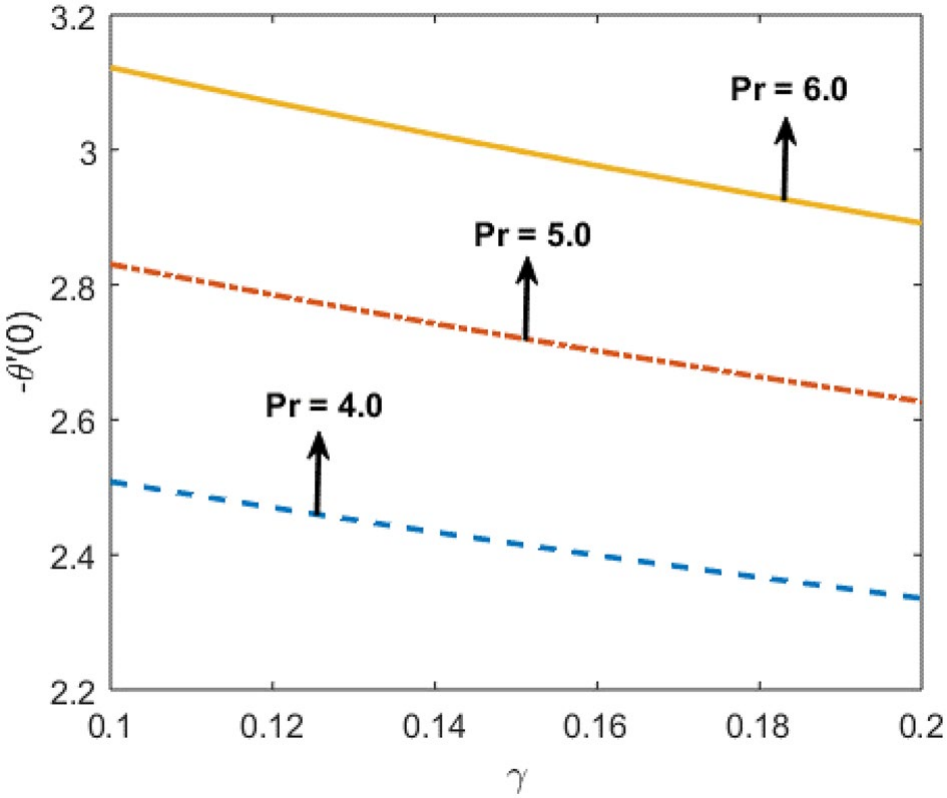
$\gamma$ and $Pr$ variations on heat transfer coefficient.

**Figure 8:**
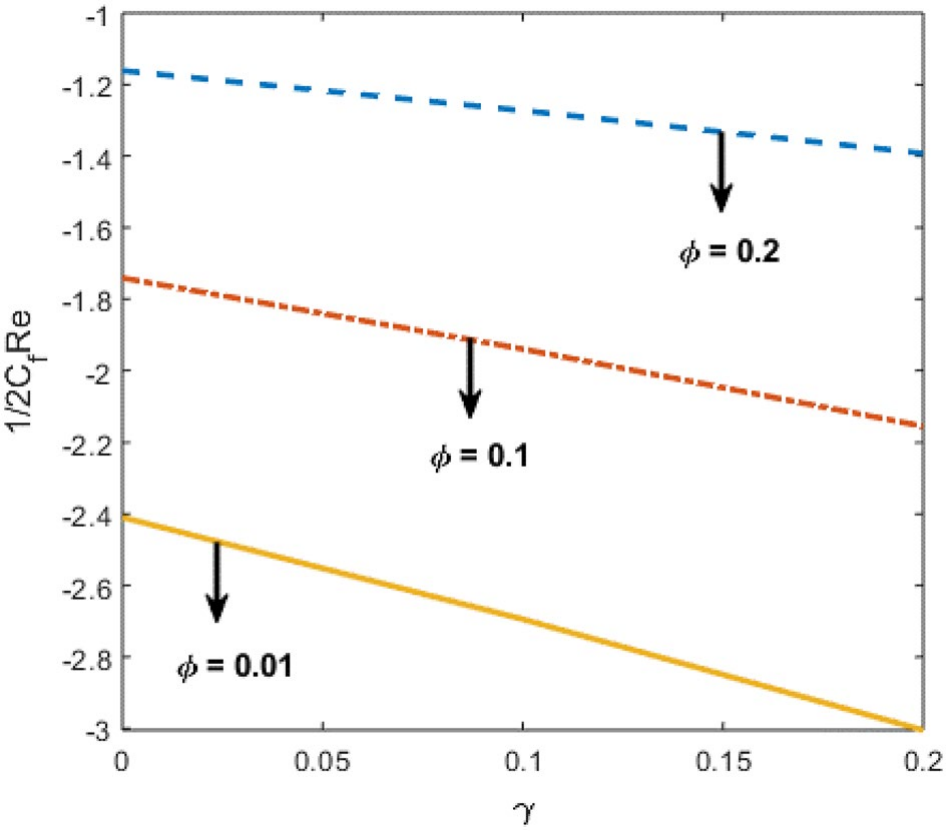
Results of $\gamma$ and $\phi$ on Skin friction coefficient.

Shah and Kumar^43^ solved the problem governing equation and plotted the expression to study the consequences of the work. The part of body that received its blood from diseased artery will not be able to get enough oxygen and energy which is important for that area of body and because of that lack the affected area cells will die in that part, causing the heart attack. Generally, it is difficult to find the analytical solution of biological phenomena presented in Fig. 1 and it shows geometrical structure of diseased artery. In results and discussion section a detail investigation of the consequences of emerging parameters is to be analyzed. Normal artery and diseased blood artery is shown in graphical abstract. Figure 2 exhibit results for the flow parameter $\gamma =0.1, 0.2, 0.3, 0.4$ impact on velocity curve. Velocity curve decreases due to increment in the value of $\gamma$. Figure 3 is plotted the outcomes of $\phi$ on curve of velocity. The velocity profile goes down owing to increment in $\phi =0.001, 0.03, 0.07, 0.2$. It is observed that due to addition of nanoparticles we can slow down the flow. Figure 4 describe the variations of temperature curve for $\gamma$. The curve goes up by increasing $\gamma =0.1, 0.2, 0.3, 0.4$. Consequences of $Pr$ on curve of temperature are shown in Fig. 5. Curve of temperature goes up by diminishing the values of $Pr=6.0, 5.0, 4.3, 3.6$. Figure 6 shows the results of $\phi$ on temperature curve. Temperature curve rises by enhancing the $\phi =0.001, 0.03, 0.07, 0.1$ values. Nusselt number variations by changing $\gamma$ and $Pr$ values are presented in Fig. 7 and the curve increases by rising $Pr$ values. Consequences of skin friction curve are shown in Fig. 8 and from results it is observed that the curve gradually goes down. Table 1 describes thermophysical properties of nanoparticles. Table 2 describes the nanoparticles and blood (base fluid) values. The results of $\gamma$ and $Pr$ on heat transfer coefficient are presented in Table 3. It is noted that the heat transfer coefficient values rises due to enhancement in $Pr$ values. Table 4 presented the results of $\gamma$ and $\phi$ on skin friction and from results it can be concluded that when solid nanoparticles volume fraction $\phi$ and $\gamma$ accelerates then the values of Skin friction coefficient also goes up.

**Table 3:** Nusselt number $-\frac{{k}_{nf}}{{k}_{f}}\theta {^{\prime}}(0)$ with respect to $\gamma$ and Pr.

$\gamma$	Pr	$-\frac{{k}_{nf}}{{k}_{f}}\theta {^{\prime}}(0)$
0.1	4.0	2.52255
0.12		2.48385
0.14		2.44730
0.1		2.52255
	5.0	2.84664
	6.0	3.13990

**Table 4:** Skin friction coefficient values with respect to $\gamma$ and $\phi .$

$\gamma$	ϕ	$\frac{1}{2}{C}_{f}Re$
0.1	0.01	− 3.12100
0.12		− 3.06967
0.14		− 3.02118
0.1		− 3.12100
	0.1	− 2.81082
	0.2	− 2.42815

## Conclusion

In present paper, we considered Newtonian characteristics of blood and the solution of the discussed problem is obtained numerically by using bvp4c. The nanofluid is considered as a mixture of copper and blood. Influence of parameters on temperature and velocity of blood is studied with the help of graphs and tables. To predict the cause of the atherosclerosis the mathematical modelling and numerical solution plays an important role and also assumed analysis summarize that nanoparticles technique could be an auspicious therapeutic approach against arterial diseases. Therefore, the present study is able to define some prime features of interest in biomedical applications. Main observations are:

1. It is observed that velocity curve decreases by increasing the values of $\gamma =0.1, 0.2, 0.3, 0.4$ and nanoparticle volume fraction $\phi =0.001, 0.03, 0.07, 0.2$.
2. Blood flow performance can be improved by managing the properties of nanoparticles.
3. As the nanoparticle concentration $\phi =0.001, 0.03, 0.07, 0.1$ increases, the temperature and flow of blood increases positively.
4. By decreasing the values of $Pr=6, 5, 4.3, 3.6$, the temperature curve increases.
5. Nusselt number graph is greatly influenced with the variation in the $Pr=4.0, 5.0, 6.0$ values.
6. Increase in nanoparticle values lessen the Skin friction curve.
7. The Cu nanoparticles as a drug are efficient to minimize the hemodynamics of stenosis.

Future application will undoubtedly concentrate on personalized medicine taking into account the specific features of individual patient.

## Data Availability

The datasets used during the current study available from the corresponding author on reasonable request.

## List of symbols

$$\left( {x,r} \right)$$: Coordinates (m)
$R$: Radius of artery (m)
$$L_{0}$$: Length (m)
$R_{0}$: Width of unblocked region (m)
${\uplambda }$: Maximum height of stenosis
$T_{0}$: Temperature of fluid ($C^{0}$)
$T, \theta$: Dimensional and dimensionless temperature ($C^{0}$)
$\phi$: Nanoparticles volume fraction
$$\psi$$: Stream function
$\nu_{f}$: Kinematics viscosity of fluid (${\text{m}}^{2} /{\text{s}}$)
${\uprho }_{f}$: Density of fluid $\left( {{\text{kg}}/{\text{m}}^{3} } \right)$
${\uprho }_{nf}$: Density of nanofluid $\left( {{\text{kg}}/{\text{m}}^{3} } \right)$
$$C_{f}$$: Skin friction
$$\eta$$: Similarity index
$$\left( {u,v} \right)$$: Velocity components (m/s)
${\upmu }_{nf}$: Viscosity of nanofluid $\left( {\text{Pa/s}} \right)$
$\mu_{f}$: Viscosity of fluid $\left( {\text{Pa/s}} \right)$
$\left( {C_{p} } \right)_{nf}$: Specific heat of nanofluid (J/kg K)
$\left( {C_{p} } \right)_{f}$: Specific heat of fluid (J/kg K)
$T_{1}$: Temperature on boundary ($C^{0}$)
${\text{k}}_{nf}$: Thermal conductivity of nanofluid $\left( {{\text{Wm}}/{\text{K}}} \right)$
${\text{k}}_{bf}$: Thermal conductivity of base fluid
$f, \epsilon$: Non-dimensional measures of stenosis
$$\gamma$$: Flow parameter
$$Pr$$: Prandtl number
$$Nu$$: Nusselt number
$\tau_{w}$: Wall shear stress
$$q_{w}$$: Heat flux
